# Life history data from the gateway to global ageing data platform: resources for studying life courses across Europe

**DOI:** 10.1007/s10433-023-00773-y

**Published:** 2023-06-21

**Authors:** Morten Wahrendorf, Christian Deindl, Jinkook Lee, Drystan Phillips

**Affiliations:** 1 Centre for Health and Society, Institute of Medical Sociology, Medical Faculty, Heinrich-Heine-University of Düsseldorf, Moorenstrasse 5, 40225 Düsseldorf, Germany; 2 Department of Social Sciences, TU Dortmund University, Dortmund, Germany; 3 Center for Economic and Social Research, University of Southern California, Los Angeles, CA USA; 4 Department of Economics, University of Southern California, Los Angeles, CA USA

**Keywords:** Life course, Life history data, SHARE, ELSA, Sequence data

## Abstract

Research from a range of disciplines highlights the need to adopt a life course perspective that considers earlier life courses to explain outcomes in later life (e.g. later life health, cognitive ageing or retirement behaviour). This includes a more comprehensive assessment of earlier life courses over time and of how they are shaped by societal and political contexts. But quantitative data with detailed information on life courses that allow to address these questions are rare. Or, in case the data are available, the data are rather difficult to handle and appears to be underused. This contribution introduces the harmonized life history data from the gateway to global ageing data platform from two European Surveys, SHARE and ELSA, with data from 30 European countries. Besides providing some details on the collection of life history data in the two surveys, we also describe the way how raw data were rearranged in a user-friendly state sequence format and additionally give some examples based on the resulting data. This illustrates the potential of collected life history data from SHARE and ELSA, clearly going beyond the description of single aspects of the life course. By providing harmonized data of two prominent studies on ageing in Europe in a user-friendly format, the gateway to global ageing data platform provides a unique data source that is easily accessible for research, and permits to study life course and their links to later life in a cross-national perspective.

## Background

Understanding how individual life courses unfold and how they may influence later life (e.g. health, economic situation, labour market participation) are central topics of various disciplines involved in ageing research, including sociology, epidemiology and psychology (Kuh et al. 2014). The interest, hereby, is not only to know whether a person was once exposed to a specific factor in a life domain, for example, if she/he worked under specific working conditions, but also to study entire trajectories, with information on the timing, the duration and the sequential character of different exposures over time, as well as the interlink of these aspects with other life domains (e.g. work-family trajectories). Another interest, hereby, is to investigate if historical, societal and political contexts shape these histories, for example, if country differences of histories exists that can be linked to different national policies. Both ideas correspond to a life course perspective and have gained increasing importance over the last decades across disciplines (Bernardi et al. 2019; Elder et al. 2003). Specifically, this concerns the idea of a holistic perspective on entire histories and the necessity to study histories in the light of their social and political contexts in which they unfold.

Quantitative data to address these topics, though, require detailed cross-national information for an extended time frame that can be linked with information on later life. This type of data is still relatively rare, and prospective cohort studies with long observation periods only partially help to overcome this shortcoming. Reasons are because some of the cohorts (especially birth cohort studies) have yet to reach older ages, or because the richness of prospective data with its information on histories is restricted to the number of waves of data collection (and the life stages covered in the data). Also, data from cohort studies are often not comparable between countries, thus preventing cross-national comparisons. An attempt to overcome these limitations is to supplement the data collection of ongoing cohort studies on ageing by retrospective interviews that additionally collect life history data. In these interviews, participants are asked about their lives before entering the study, for example, entire employment histories the respondent previously had.

An increasing number of studies conduct such life history interviews, including two significant studies of ageing in Europe ageing: the English Longitudinal Study of Ageing (ELSA) (Steptoe et al. 2013), and the Survey of Health, Ageing and Retirement in Europe (SHARE) (Börsch-Supan et al. 2013a). Both studies are part of the international family of Health and Retirement studies around the world, which are all developed in close coordination, with a focus on harmonizing research methods and study designs to allow for cross-national comparisons. Importantly, both studies not simply collect life history data through structured questions as part of their face-to-face interview. Instead, based on advances in research on autobiographical memories (for an overview see: Smith et al. 2021), the collection of data occurs with the help of a graphical representation of the life course (or a “calendar”). This calendar is visible to participants on a computer screen in the interview and consists of a two-dimensional grid, where the x-axis describes the temporal dimension (e.g. years), and the y-axis different domains of the life course (e.g. children, employment or housing). When filling this calendar during the interview, respondents have the possibilities to cross-reference between different histories (e.g. job when first child was born), as well as major “landmark events” are provided (e.g. year of moon landing) that help to memorize the life course. Surely, recall bias remains an issue, and some information (e.g. attitudes and beliefs questions) are difficult to be asked retrospectively. But there is widespread consensus that calendar interviews contribute to better quality and more accurate retrospective information (Axinn et al. 1999; Belli 1998; Belli et al. 2007; Blane 1996; Drasch & Matthes 2011; Freedman et al. 1988), for example, when asking about socio-demographic conditions or employment histories (Baumgarten et al. 1983; Wahrendorf et al. 2019). Also, compared with prospective data collection (where question wording and order in the interview may change across waves of data collection), retrospective data make sure that information (referring to different time points) is equally assessed.

Life history data have now been collected for nearly 100,000 respondents across Europe as part of SHARE and ELSA. Thereby, the domains or different topical areas covered across the life course range from family life and partnership relationships, housing histories and geographical mobility, to employment histories (including paid work and home or family work) to health histories with information on major periods of poor health or disability. This provides remarkable opportunities for comparative life courses research, specifically, for studying previous life courses in a cross-country perspective including the association between life course factors (e.g. patterns of family formation) and various later life outcomes (e.g. health, later life cognitive functioning, retirement behaviour, financial situation). However, albeit the data are freely available for research purposes, the richness and full potential of the data are still not fully exploited. One important reason may be the difficulty associated with learning multiple surveys, and—specifically in case of life history data—the complex data structure resulting from life history interviews (see below for details). This structure requires extensive data management in each survey to provide information on entire histories, that allow to fully exploit the longitudinal nature of the data.

For these reasons, the gateway to global ageing data (sponsored by the National Institute on Aging) was developed as a platform to make data more accessible to researchers and to facilitate cross-country analyses among surveys of ageing, especially those using the Health and Retirement Study (HRS) and its international sibling studies around the globe. One major achievement of the platform, hereby, is to provide harmonized datasets from participating surveys, accompanied by extensive documentations of questionnaires and the provision of all codes to create these harmonized data (Lee et al. 2021). Such harmonized data have now also been developed for the life history data from SHARE and ELSA, and—in contrast to the raw data provided by the single surveys—were released in a user-friendly sequence data format (see below for details).

In this article, we introduce the harmonized life history data from SHARE and ELSA provided by the gateway to global ageing data platform, and give some illustrative examples of resulting data to show its potential for life courses research.

## Life history data in SHARE and ELSA

ELSA began in 2002 in England (not covering the entire UK), and SHARE in 2004, with ongoing waves of data collection in two-year intervals and new respondents (so-called “refreshers”) being added subsequently to maintain population representation in both surveys. The two studies rely on nationally representative samples of individuals aged 50 and older (based on probability household samples where respondents and partners are interviewed). This corresponds to a target population of persons born in 1952 or earlier for ELSA and in 1954 or earlier for SHARE (in the first waves, respectively). While SHARE started in 12 countries (11 European countries and Israel), new countries joined SHARE in the subsequent years, with 29 participating countries since study onset. For detailed data resource profiles of ELSA, see (Steptoe et al. 2013), and for SHARE (Börsch-Supan et al. 2013a).

In addition to regular waves which focus on current life circumstances of participants at moment of data collection, both surveys also had life history interviews. In ELSA, this was firstly conducted between March and October 2007 (in addition to the core interview in wave 3), and in SHARE between autumn 2008 and summer 2009 (as a separate life history interview in place of a core interview, often also referred to as “SHARELIFE”) (Börsch-Supan et al. 2013b). In addition, wave 7 of SHARE repeated the life history survey for all respondents (and countries) who were not part of wave 3. More details can be found in the respective methodological volumes of SHARE (Bergmann et al. 2019; Schröder 2011) and ELSA (Ward et al. 2009). A number of completed life history interviews in the two studies, including mean age and sex distribution for each country, are presented in Table 1. In sum, comparable life history data exist for 99,559 respondents from 30 countries across Europe, where most histories were conducted in wave 7 of SHARE.

**Table 1:** Completed life history interviews for the harmonized data from the gateway to global ageing data, incl. mean age and sex distribution

Survey	Country	Wave 3				Wave 7				Total
		No	Age	Sex %		No	Age	Sex %		
			(Mean)	Male	Female		(Mean)	Male	Female	No
*ELSA*	England	7855	(66.7)	46.7	53.3	–	–	–	–	7855
*SHARE*	Austria	994	(66.2)	45.0	55.0	2693	(66.8)	46.3	53.7	3687
	Germany	1918	(66.2)	45.7	54.3	2982	(66.7)	46.7	53.3	4900
	Sweden	1961	(66.4)	47.3	52.7	2129	(67.0)	48.1	51.9	4090
	Netherlands	2258	(64.9)	47.2	52.8	–	–	–	–	2258
	Spain	2271	(66.6)	45.8	54.2	3424	(67.4)	46.3	53.7	5695
	Italy	2528	(66.3)	45.2	54.8	3000	(66.7)	45.8	54.2	5528
	France	2500	(66.2)	45.1	54.9	2186	(66.6)	45.6	54.4	4686
	Denmark	2144	(65.1)	47.2	52.8	1961	(66.1)	47.9	52.1	4105
	Greece	3090	(66.4)	46.4	53.6	1160	(67.1)	45.9	54.2	4250
	Switzerland	1324	(65.6)	46.3	53.7	1648	(66.4)	47.4	52.6	2972
	Belgium	2865	(65.9)	46.0	54.0	3333	(66.5)	46.8	53.2	6198
	Israel	–	–	–	–	2131	(65.3)	46.4	53.6	2131
	Czechia	1816	(64.4)	44.9	55.1	3292	(65.9)	45.6	54.4	5108
	Poland	1939	(63.8)	43.5	56.5	3559	(65.1)	44.1	55.9	5498
	Ireland	855	(65.9)	48.0	52.0	–	–	–	–	855
	Luxembourg	–	–	–	–	1250	(65.2)	48.5	51.5	1250
	Hungary	–	–	–	–	1538	(66.7)	42.4	57.6	1538
	Portugal	–	–	–	–	1282	(68.2)	44.4	55.6	1282
	Slovenia	–	–	–	–	3691	(66.0)	46.3	53.7	3691
	Estonia	–	–	–	–	5115	(66.7)	40.1	60.0	5115
	Croatia	–	–	–	–	2408	(66.1)	44.5	55.5	2408
	Lithuania	–	–	–	–	2035	(65.9)	39.7	60.3	2035
	Bulgaria	–	–	–	–	1998	(66.0)	44.4	55.6	1998
	Cyprus	–	–	–	–	1233	(65.4)	47.8	52.2	1233
	Finland	–	–	–	–	2007	(66.3)	46.4	53.6	2007
	Latvia	–	–	–	–	1734	(66.4)	39.0	61.0	1734
	Malta	–	–	–	–	1261	(65.8)	47.6	52.4	1261
	Romania	–	–	–	–	2114	(66.1)	44.4	55.6	2114
	Slovakia	–	–	–	–	2077	(64.4)	44.3	55.7	2077
Total		36,318				63,241				99,559

ELSA = English Longitudinal Study on Ageing; SHARE = Survey of Health, Ageing and Retirement in Europe

Values for mean age and sex proportions are based on weighted data and use latest version of SHARE (version B.3) and ELSA (version A.2) of the Gateway to Global Aging Data platform

## Data structure and domains covered

As part of the life history survey respondents provided information for five domains: employment, partnership, children, health and accommodation. As an example, in the case of employment histories, all respondents gave information on each paid job they had till the moment of the interview and details for periods when they were not in paid employment (e.g. gap between two jobs). For each of these job spells (or spells when not working), the data contain information on the year when the respective spell started and ended, and variables that specify the spell (e.g. in self-employment, doing home or family work, or retired). The same type of information was collected for accommodation spells or for partnerships. Based on this “spell data format”, though, it is quite complicated for non-experienced researcher to derive simple statistics, for example, to know what the exact employment situation of a person was at age 40. For some respondents, this information may be stored in the second job spell—and for others the information may be given in the provided details for the period when not working between the second and the third job. Technically, this requires extensive data management where the provided spell information of each individual (with differing numbers of spells between individuals) is used to ascertain a specific state at a given age (based on a defined list of possible states). Further readings with additional information on spell data, its management and analyses can be found elsewhere (Blanchard et al. 2014; Kröger 2015; Ritschard & Studer 2018).

Therefore, to facilitate the use of life history data, the gateway provides harmonized life history data in a state sequence format, where—for each domain covered in the interview—data are rearranged in a less complex and more intuitive state sequence format. This means that the datasets contain a discrete state variable for each age covered in the life history interview to describe the state at a particular age (e.g. the work situation at the age of 20, 21, 22, 23, 24, etc.). As an example, the variable “WORKSTATE25” captures the employment situation at age 25, and the variable “WORKSTATE50” the employment situation at age 50. As a consequence, for each individual who completed a life history interview, this provides information on whole sequences, with information on successive states throughout the life course. Table 2 illustrates the information evolving from these data using employment histories between age 15 and 65 as an example (covering 51 years). Each history is presented as a sequence of letters where the letter represents a specific employment situation.

**Table 2:** Examples of employment sequences from age 15 to 65

Employment sequences	
EEEEWWWWWWWWWWWWWWWWWWWWWWWWWWWWWWWWWWWWWWWWWWRRRRR	
EEEESSSSSSSSSSSSSSSSSSSSSSSSSSRRRRRRRRRRRRRRRRRRRRR	
EEEEEEEEEEWWWHHHHHHHHHHHHwwwwwwwwwwwwwwwwwwwwRRRRRR	
EEEEEEEEEWWUUUWWWWUWWWWUwwwwwwwwWWWWWWWWWWWWWRRRRRR	

E = "Full-time education”, W = "Employed full-time”, w = "Employed part-time”, S = "Self-employed”, U = "Unemployed”, H = "Home/family work”, R = "Retired”, O = "Other”

The first two sequences belong to persons who started to work full-time after full-time education at the age of 19, either in full-time employment (person 1) or as self-employment (person 2), and were both constantly working till entering retirement, with comparatively early retirement of the second person. The remaining examples belong to persons who started working somewhat later after full-time education, and either had a long period of home or family work that was followed by part-time work (person 3), or repeated unemployment interruptions (person 4).

Taken together, the state sequence format directly allows to describe the circumstance at a specific age (without need of extensive data management). And this format is also the standard format for sequence analyses (Halpin 2017; Ritschard and Studer 2018; Studer 2013). Sequence analyses allow a comprehensive analyses of whole sequences of respondents. This ranges from the creation of summary measures of entire histories, such as years spent in a specific state (e.g. in paid work) or the identification of specific pattern of interest (e.g. reemployment after long period of unemployment), to techniques that allow to regroup similar histories into typologies.

Full details on the procedures to re-arrange the data and on the derived harmonized states for each of the five histories in the two surveys are available online in the respective codebooks of the two studies (Wahrendorf et al. 2023a, 2023b). These also contain details on the variables used in the raw datasets, on the naming conventions of the created variables, and how it was ensured that identical concepts and questions were used when harmonizing the data. An overview of the resulting histories and the derived harmonized states for each of the five histories is provided in Table 3.

**Table 3:** Domains of the harmonized histories

Domain	Annual information from age 15 to age 80 on…
*Work*	Different main work states, including paid work, unpaid work and states when not working (up to 8 categories)
*Partnership*	Whether respondent lived with a partner (irrespective of marital status)
*Children*	Total number of children (regardless of age of children), and total number of children below age 18 (counting biological and adopted children in both cases)
*Housing*	Different housing states, including private (owner or tenant), non-private housing, living abroad or still living in parental home (5 categories)
*Health*	Whether had period of ill health or disability (not specifying type of illness)

### Access to data and documentation via the gateway

To access the data, registered users can either download the generated datasets or the programs that will generate the harmonized dataset from the study's raw data files. Specifically, for ELSA the created harmonized dataset can be downloaded from the UK data repository (without need to run the creation code). For SHARE, users need to download the raw data from the SHARE Research data centre and run the creation code in Stata that is available on the gateway. All necessary information, sources or links to respective data sources are provided on the gateway. The generated dataset also contains an ID-variable that allow to merge life history data with other datasets of the respective study, for example, with health data from the remaining waves of data collection. Furthermore, the data contain study-specific sampling weights for respondents of the life history interviews (referring to moment of data collection), together with stratum and cluster variables that account for complex survey design.

### Some illustrative results

In the following, we give two simple examples of the collected life history data (for illustrative purposes without intention of an in-depth analyses of the addressed topic). All figures are based on the latest release of the gateway (based on SHARE version B.3 & ELSA version A.2) and have been produced with Stata. We apply weights to produce appropriate population estimates, and also account for different sample sizes of each country when estimating proportions across countries.

**Fig. 1:**
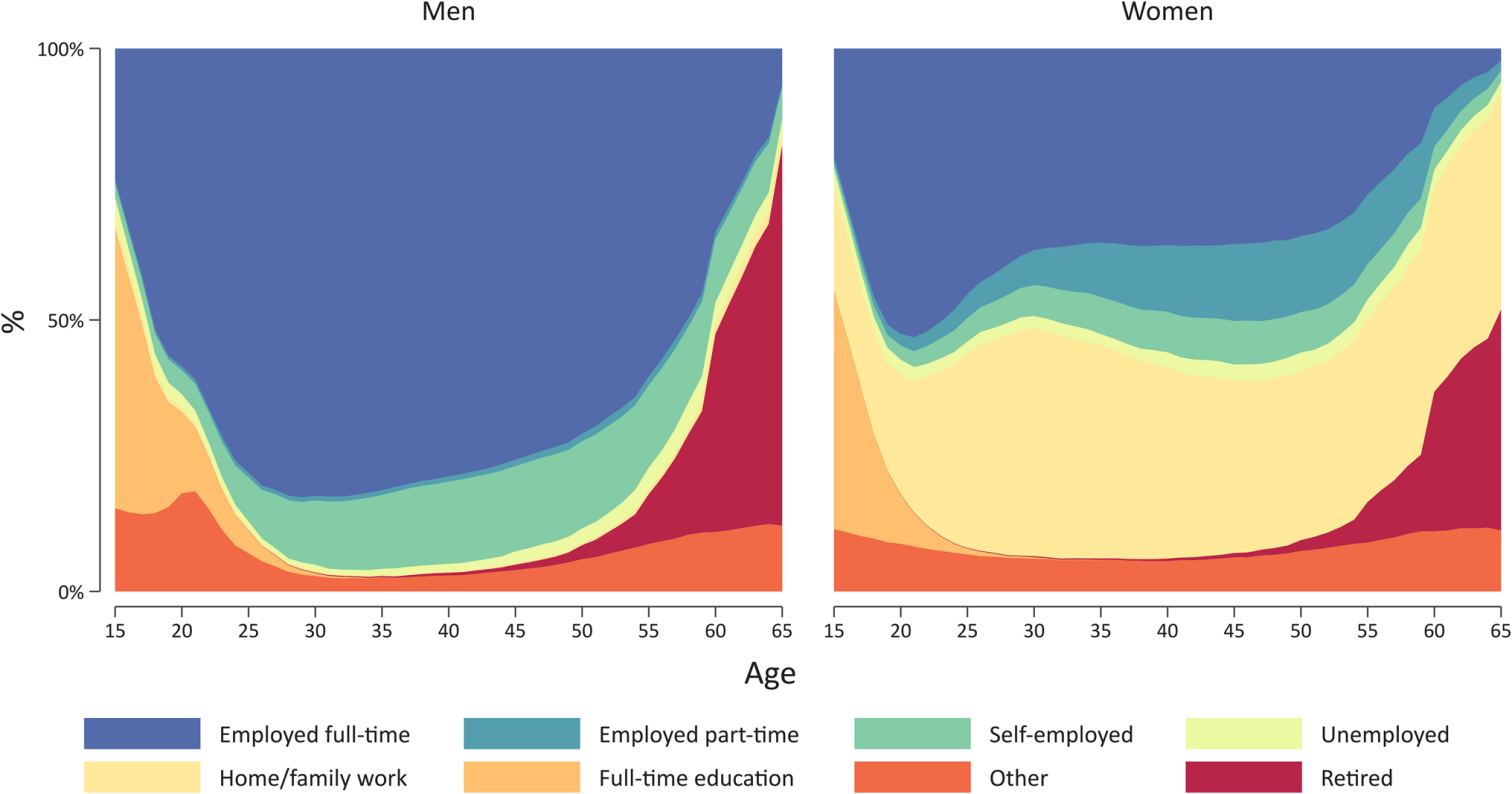
Chronogram of employment histories by sex, based on annual data of the employment situation between age 15 and 65 (15,939 men and 20,379 women). Note: Values are based on weighted data that (also accounting for different sample sizes of each country and use wave 3 data of the latest Version of SHARE (Version B.3) and ELSA (Version A.2) of the Gateway to Global Aging Data platform

**Fig. 2:**
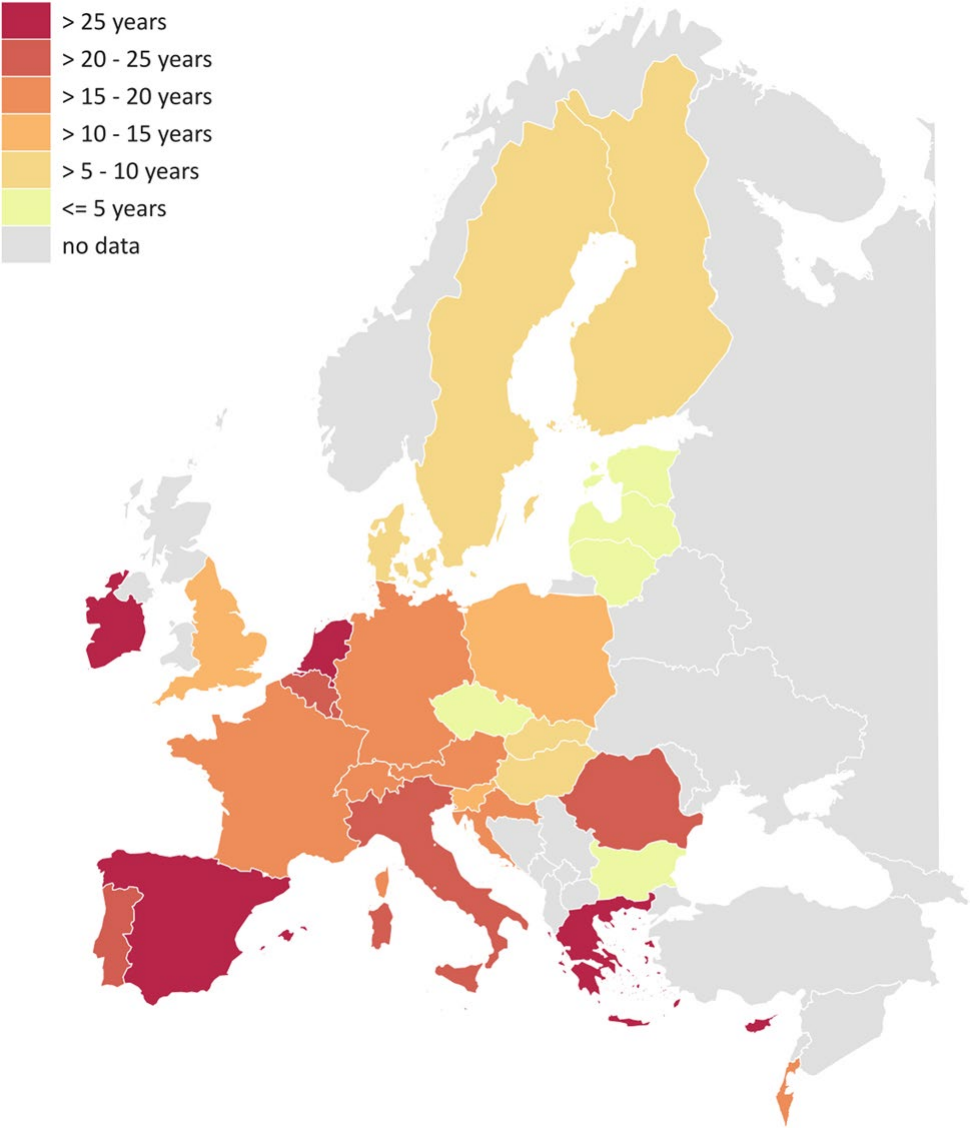
Average years spent in home/family work as main employment situation between age 15 and age 65 for women, based on employment histories of 26,215 women (aged 65 or older). *Note*: Values are based on on weighted data and use wave 3 and wave 7 data of latest Version of SHARE (Version B.3) and ELSA (Version A.2) of the Gateway to Global Aging Data platform

Figure 1 presents the observed distribution of eight different employment states of the harmonized employment histories between age 15 and 65 years, separately for men and women across both surveys. We clearly see how the proportions of some states vary across the life course, for example, that the proportion of respondents who were in full-time education is highest at the beginning of the observation period and quickly declines thereafter. Also, there are differences between men and women, with higher labour market participation (mostly full-time employment) for men compared with women. Another finding is that there is almost no home or family work for men, and that women spent a large amount of their employment histories in home or family work. Whether the average time (measured in years) spent in home or family work for women (again for the period between age 15 and 65) varies by country is shown in Fig. 2. With average scores above 25 years, highest values are found in the Mediterranean countries Spain, Greece, Malta and Cypress, together with Ireland and the Netherlands. In contrast, values are clearly lower in the Nordic countries Denmark, Sweden and Finland as well as Hungary and Slovakia, but especially in the three Baltic countries (Estonia, Latvia and Lithuania) together with Bulgaria and the Czech Republic (with less than 5 years). These results clearly suggest that employment histories vary between men and women, and also indicate how women’s situation in the paid labour market and the gender division of labour are very different across Europe. This has potentially important impact on later economic situations and health of older persons [for an example linking employment histories with later health or cognitive functioning see (Wahrendorf et al. 2021) or (Ice et al. 2020)]. And possibly these patterns are related to different social and labour market policies, including childcare regulations or different family policies. The two figures give two simple examples for potential sex and country differences in employment histories that are probably linked to the wider sociopolitical contexts.

## Discussion

The presented harmonized life history data of SHARE and ELSA from the gateway to global ageing data platform provides remarkable opportunities for studying and comparing previous life course across Europe, as well to study their links with later life outcomes. Besides being delivered in a harmonized format (and thus allowing for cross-national comparisons), one key feature of the data is that they are delivered in a user-friendly state sequence format that make data more accessible for the research community and allows an in-depth analyses of entire histories. On a conceptual level, this means that the data may facilitate researchers to address one core idea of the life course perspective when studying life course in Europe (Bernardi et al. 2019; Elder et al. 2003), namely, the necessity of studying histories as a whole. Importantly, while the two presented studies are two significant studies of ageing in Europe a, an additional life history dataset is already available from the China Health and Retirement Longitudinal Study (CHARLS) (Wahrendorf et al. 2022), and in progress from HRS in the US. Also, it is worth noting at this point that SHARE and ELSA also ask specific questions about childhood conditions (i.e. childhood health and socioeconomic conditions at age 10). These childhood data are also incorporated into the Harmonized datasets of the gateway, and—albeit referring to a single time point only—is surely of interest for life course researcher interested in consequences of early-life conditions.

Some limitations of the harmonized data, though, must be named: First, when harmonizing life history data, it is inevitable that some details are not used. As an example, some researchers may be interested in intra-generational social mobility processes when studying employment histories. Yet, while SHARE collected information on the occupation respondents had (first digit of the ISCO code), ELSA did not ask for this aspect. In case of interest, users may therefore modify and adapt the provided do-files of SHARE to their own purposes. Second, when creating the state sequence data, some decisions are needed, for example, to decide which state is prioritized in the unlikely case that respondents reported both paid employment and a period of not working. For these details, we refer people to the codebook, where respective strategies are clearly described. Third, in the case of health history, the available information (i.e. period of ill health or not) must be considered as rather limited, specifically if researchers are interested in single diseases and the age of diagnoses (e.g. cardiovascular diseases or symptoms of depression). In that case we recommend to use data from core waves where different diseases and their age at onset are assessed, thus allowing to investigate life time prevalence of specific diseases or the age when a disease was diagnosed. Fourth, because of legal regulations, the gateway cannot directly release the harmonized data files on its website, and therefore, needs to refer to other sources. Future users, though, may profit from a gateway data enclave that is in progress, where users will be able to access data from a virtual gateway without need to go through the respective provider of survey data.

In conclusion, albeit life history data are increasingly available in ongoing studies on ageing, the extent to which the potential is used is still very restricted. By providing harmonized data of two prominent studies on ageing in Europe in a user-friendly format, we hope this facilitates its use and stimulates research in the field.

## References

[CR1] Axinn WG, Pearce LD, Ghimire D (1999). Innovations in life history calendar applications. Soc Sci Res.

[CR2] Baumgarten M, Siemiatycki J, Gibbs GW (1983). Validity of work histories obtained by interview for epidemiologic purposes. Am J Epidemiol.

[CR3] Belli R (1998). The structure of autobiographical memory and the event history calendar: potential improvements in the quality of retrospective reports in surveys. Memory.

[CR4] Belli R, Smith LM, Andreski PM, Agrawal S (2007). Methodological comparisons between CATI event history calendar and standardized conventional questionnaire instruments. Public Opin Q.

[CR5] Bergmann M, Scherpenzeel A, Börsch-Supan A (2019) SHARE Wave 7 methodology: panel innovations and life histories. Munich: MEA, Max Planck Institute for Social Law and Social Policy

[CR6] Bernardi L, Huinink J, Settersten RA (2019). The life course cube: a tool for studying lives. Advances in Life Course Research.

[CR7] Blanchard P, Bühlmann F, Gauthier J-A (2014) Advances in sequence analysis: theory, method, applications (vol 2): Springer

[CR8] Blane DB (1996). Collecting retrospective data: development of a reliable method and a pilot study of its use. Soc Sci Med.

[CR9] Börsch-Supan A, Brandt M, Hunkler C, Kneip T, Korbmacher J, Malter F (2013). Data resource profile: the survey of health, ageing and retirement in Europe (SHARE). Int J Epidemiol.

[CR10] Börsch-Supan A, Brandt M, Schroder M (2013). SHARELIFE-one century of life histories in Europe. Adv Life Course Res.

[CR11] Drasch K, Matthes B (2011) Improving retrospective life course data by combining modularized self-reports and event history calendars: experiences from a large scale survey. Qual Quant 47(2):817–838. 10.1007/s11135-011-9568-0

[CR12] Elder GH, Johnson MK, Crosnoe R, Mortimer JT, Shanahan MJ (2003). The emergence and development of life course theory. Handbook of the life course.

[CR13] Freedman D, Thornton A, Camburn D, Alwin D, Young-demarco L (1988). The life history calendar: a technique for collecting retrospective data. Sociol Methodol.

[CR14] Halpin B (2017). SADI: sequence analysis tools for Stata. Stata J.

[CR15] Ice E, Ang S, Greenberg K, Burgard S (2020). Women’s work-family histories and cognitive performance in later life. Am J Epidemiol.

[CR16] Kröger H (2015). newspell: easy management of complex spell data. Stand Genomic Sci.

[CR17] Kuh D, Cooper R, Hardy R, Richards M, Ben-Shlomo Y (2014) A life course approach to healthy ageing (First edition. ed., Life course approach to adult health series). Oxford, UK; New York, United States of America: Oxford University Press

[CR18] Lee J, Phillips D, Wilkens J, Team GTGAD (2021) Gateway to global aging data: resources for cross-national comparisons of family, social environment, and healthy aging. J Gerontol: Ser B 76(Supplement_1):S5–S16 10.1093/geronb/gbab050 PMC8186854 33861849

[CR19] Ritschard G, Studer M (2018) Sequence analysis and related approaches: innovative methods and applications: Springer

[CR20] Schröder M (2011) Retrospective data collection in the survey of health, ageing and retirement in Europe. SHARELIFE Methodology. Mannheim: Mannheim Research Institute for the Economics of Aging

[CR21] Smith J, Hu M, Lee H, Ferraro KF, Carr D (2021). Chapter 3: Measuring life course events and life histories. Handbook of aging and the Social Sciences.

[CR22] Steptoe A, Breeze E, Banks J, Nazroo J (2013) Cohort profile: the English longitudinal study of ageing. Int J Epidemiol 1640–1648. 10.1093/ije/dys168 10.1093/ije/dys168 PMC3900867 23143611

[CR23] Studer, M. (2013). WeightedCluster library manual: a practical guide to creating typologies of trajectories in the social sciences with R. In: LIVES working papers, 24, 1–34

[CR24] Wahrendorf M, Deindl C, Beaumaster S, Phillips D, Lee J (2023a) Harmonized ELSA life history documentation , version A.2. Los Angeles, CA: Center for Economic and Social Research, University of Southern California

[CR25] Wahrendorf M, Deindl C, Beaumaster S, Phillips D, Lee J (2023b) Harmonized SHARE life history documentation, Version B.3. Los Angeles, CA: Center for Economic and Social Research, University of Southern California

[CR26] Wahrendorf M, Deindl C, Phillips D, Lee J (2022). Harmonized CHARLS life history documentation, version A.

[CR27] Wahrendorf M, Hoven H, Deindl C, Lunau T, Zaninotto P (2021) Adverse employment histories, later health functioning and national labor market policies: European findings based on life-history data From SHARE and ELSA. J Gerontol. Ser B: Psychol Sci Soc Sci 76(Supplement_1):S27–S40. 10.1093/geronb/gbaa049 10.1093/geronb/gbaa049 PMC8495751 32322883

[CR28] Wahrendorf M, Marr A, Antoni M, Pesch B, Jockel KH, Lunau T (2019). Agreement of self-reported and administrative data on employment histories in a German Cohort Study: a sequence analysis. Eur J Popul.

[CR29] Ward K, Medina J, Mo M, Cox K (2009). ELSA wave three: life history interview: a user guide to the data.

